# Improvement in Functional Properties of Soy Protein Isolate-Based Film by Cellulose Nanocrystal–Graphene Artificial Nacre Nanocomposite

**DOI:** 10.3390/polym9080321

**Published:** 2017-07-30

**Authors:** Kuang Li, Shicun Jin, Yufei Han, Jianzhang Li, Hui Chen

**Affiliations:** 1 Key Laboratory of Wood Materials Science and Utilization (Beijing Forestry University), Ministry of Education, Beijing 100083, China; kuangli@bjfu.edu.cn (K.L.); jinsc1994@bjfu.edu.cn (S.J.); hanyufei@bjfu.edu.cn (Y.H.); 2 Beijing Key Laboratory of Wood Science and Engineering, Beijing Forestry University, Beijing 100083, China; 3 College of Materials Science and Technology, Beijing Forestry University, Beijing 100083, China

**Keywords:** soy protein isolate, graphene, cellulose nanocrystal, artificial nacre, nanocomposite film

## Abstract

A facile, inexpensive, and green approach for the production of stable graphene dispersion was proposed in this study. We fabricated soy protein isolate (SPI)-based nanocomposite films with the combination of 2D negative charged graphene and 1D positive charged polyethyleneimine (PEI)-modified cellulose nanocrystals (CNC) via a layer-by-layer assembly method. The morphologies and surface charges of graphene sheets and CNC segments were characterized by atomic force microscopy and Zeta potential measurements. The hydrogen bonds and multiple interface interactions between the filler and SPI matrix were analyzed by Attenuated Total Reflectance–Fourier Transform Infrared spectra and X-ray diffraction patterns. Scanning electron microscopy demonstrated the cross-linked and laminated structures in the fracture surface of the films. In comparison with the unmodified SPI film, the tensile strength and surface contact angles of the SPI/graphene/PEI-CNC film were significantly improved, by 99.73% and 37.13% respectively. The UV–visible light barrier ability, water resistance, and thermal stability were also obviously enhanced. With these improved functional properties, this novel bio-nanocomposite film showed considerable potential for application for food packaging materials.

## 1. Introduction

Biodegradable and sustainable biopolymers derived from natural resources, such as proteins, cellulose, lignin and polysaccharides, have aroused considerable interest in developing alternatives to petroleum-derived plastics [1,2,3,4,5]. In comparison with other natural biopolymers, soy protein isolate (SPI) derived from plant proteins has been the focus of much research owing to its advantages of low cost, biodegradability, biocompatibility, and easy availability [6,7,8,9]. These notable advantages make it an ideal material for various applications in packaging materials [10], filtration membranes [11], drug delivery systems [12], and tissue engineering [13]. However, the natural defects of SPI-based films, such as inferior mechanical properties and moisture sensitivity, limit their practical application. Many methods have been applied to improve the properties of SPI-based materials, including physical treatment [14], enzyme treatment [15], and block copolymerization [16]. Nanophase reinforcement is one of the most effective methods for enhancing the performance of polymer composites and adhesives [17]. A critical merit of functional nanocomposite is its ability to maximize stress transfer across interfaces in order to improve the strength and toughness of the materials [18]. However, weak interfacial interactions and poor dispersion of reinforcing components may lead to brittle materials with limited mechanical properties. To tackle these challenges, much attention has been paid to the development of layered nanomaterials as fillers in the polymer composites [19].

Nacre, a ‘‘brick and mortar’’ layered architecture comprised of 95 vol % two-dimensional (2D) aragonite calcium carbonate platelets and 5 vol % one-dimensional (1D) nanofibrillar chitin and protein, has been a new source of inspiration for designing many synthetic hybrid composites and nanomaterials [20]. The highly ordered 2D components, combined with hydrogen bonding and dense covalent with biopolymer, contribute to highly effective load transfer between the inorganic platelets and organic protein, resulting in high strength and toughness [18]. Among 2D reinforcing nanomaterials, graphene is considered as a carbon nanofiller that consists of sp^2^-hybridized carbon atoms with a one-atom-thick, 2D honeycomb hexagonal structure, which has emerged as a functional nanomaterial in the preparation of high-performance polymer nanocomposites [21]. Because of its excellent mechanical properties, high thermal conductivity, and superior chemical stability, graphene has attracted considerable attention in multidisciplinary research fields and shows great potential in various applications [22,23]. Cellulose nanocrystals (CNC) are rod-shaped or whisker shaped highly crystalline nanosize segments that can be obtained from natural cellulose fibers with eliminated amorphous regions by controlled acid hydrolysis [24]. The high aspect ratio, high specific strength, and nanometric dispersion make CNC an appropriate and ideal candidate as an effective reinforcing nanofiller for different polymer composites [25]. CNC possesses a highly ordered crystalline structure and negatively charged sulfate ester groups through the sulfuric acid hydrolysis process [26].

**Scheme 1:**
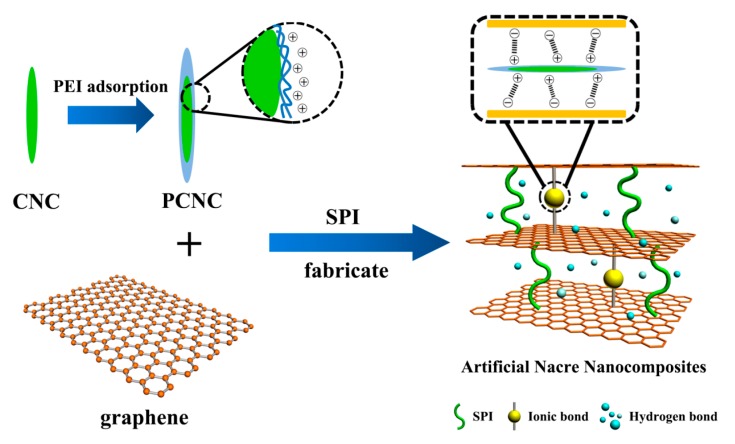
Schematic illustration of the soy protein isolate (SPI)-based nanocomposite film.

Here, we proposed a facile, inexpensive, and green approach to preparing stable aqueous graphene dispersions via sonication in bovine serum albumin (BSA) solution. A layer-by-layer (LbL) assembly technique was adopted to prepare SPI-based nanocomposite films. We assembled the strength and flexible 2D graphene sheets with a high aspect ratio 1D CNC component to produce active interfacial adhesion laminated nanocomposites. Due to the fact that CNC was prepared by the sulfuric acid hydrolysis method and possessed negatively charged sulfate ester groups, we modified the CNC with the cationic polyethyleneimine (PEI) to introduce positive charged surface functionalities via strong ion pairing with anionic graphene sheets to enhance the interfacial interactions among these components (Scheme 1). This strongly positively charged CNC could facilitate the strong ionic interactions with negatively charged graphene sheets for the effective dispersion and LbL assembly. Therefore, this novel SPI-based nanocomposite film exhibited enhancements in mechanical properties as well as water resistance. The microstructure, surface hydrophilicity, and thermostability of this composite film were also investigated.

## 2. Materials and Methods 

### 2.1 Materials

SPI (95% protein) was acquired from Yuwang Ecological Food Industry Co., Ltd. (Shandong, China). Graphite powder was purchased from Sinopharm Chemical Reagent Co., Ltd. (Beijing, China). BSA (98% purity) was provided by Amresco (Solon, OH, USA). Microcrystalline cellulose [MCC, (C_6_H_10_O_5_)*_n_*] was obtained from Sinopharm Chemical Reagent Co., Ltd. (Beijing, China). Polyethyleneimine (PEI) was provided by Tianjin Heowns Biochem Co., Ltd. (Tianjin, China). Glycerol (99% purity), Hydrogen Chloride, sodium hydroxide, and other chemical reagents were analytical grade and provided by Beijing Chemical Reagents Company (Beijing, China). Deionized water was used for the preparation of all aqueous solutions.

### 2.2 Preparation of Aqueous Graphene Dispersion

The first step was to disperse BSA powder (50 mg) in 100 mL deionized water and stir constantly at 50 °C for 2 h. Next, graphite powder (1 g) was added into 200 mL well-dispersed BSA solution and ultrasound-treated while being stirred for 30 min. The pH of the mixture was adjusted to 3.0 with HCl. Finally, the sonicated mixture was centrifuged at 4000 rpm for 30 min to obtain dispersion of graphene in the suspension.

### 2.3 Fabrication of CNC and PEI-Modified CNC Colloidal Suspensions

MCC (10 g) was dispersed in 200 mL of H_2_SO_4_ (64 wt %) at 45 °C under constant stirring for 60 min. The mixture was diluted with deionized water and centrifuged at 12,000 rpm for 10 min. Next, the suspension was dialyzed against deionized water until the wash water maintained at constant pH. The samples were sonicated for 30 min to obtain stable CNC colloidal suspensions

PEI-modified CNC colloidal suspensions were prepared by the following procedure: first, 50 mL of CNC suspension (1 wt %) was mixed with 100 mL of PEI (1 wt %) under constant stirring. The pH of mixture was adjusted to 1.5 with HCl (5 wt %) to promote ionic interactions between CNC and PEI. Then, the mixture was centrifuged at 14000 rpm for 10 min and washed with deionized water to remove free PEI. Finally, the PEI-modified CNC suspension was diluted to 1 wt %.

### 2.4 Preparation of Graphene/CNC Modified SPI-Based Films

SPI-based films were prepared by a casting method. First, SPI powder (5 g) and glycerol (2.5 g) were dispersed in 95 g of deionized water or prepared graphene dispersion with magnetic stirring for 30 min. The pH of the mixture was adjusted to 9.0 with NaOH solution (10%). Then, 2 g of CNC or PEI-modified CNC suspension (1 wt %) was dispersed in the SPI solution and stirred constantly at 85 °C for 30 min. After that, the mixture was ultrasound-treated for 10 min to remove the bubbles in solution. Finally, the mixture was poured into Teflon-coated plates and dried in a vacuum oven at 45 °C for 24 h. All films were placed in a desiccator at 25 °C and 50% relative humidity (RH) with saturated K_2_CO_3_ solution for 48 h before testing. The forming mechanism of the nanocomposite film is depicted in Scheme 1.

### 2.5. Characterization

#### 2.5.1. Atomic Force Microscopy

Atomic force microscopy (AFM, Multimode 8, Bruker, Billerica, MA, USA) was used to characterize the surface morphology of graphene sheets, CNC, and the PEI-modified CNC monolayer.

#### 2.5.2. Zeta Potential Measurements

The charge on the surface of graphene sheets and CNC suspensions was characterized by SZ-100nanopartica series instruments (Horiba, Kyoto, Japan) by measuring the zeta potential of a suspension. The sample was injected into a disposable cell and a measurement of the particle electrophoretic mobility resulted in the calculated zeta potential.

#### 2.5.3. Structural Analysis

Structural characteristics of SPI-based nanocomposite films were analyzed by Attenuated Total Reflectance–Fourier Transform Infrared (ATR–FTIR) spectra and X-ray diffraction (XRD) patterns. ATR–FTIR spectra were recorded by a Nicolet 6700 spectrometer (Thermo Scientific, Madison, WI, USA) in the wave number range of 600–4000 cm^−1^ with 32 scans. XRD patterns were conducted with a D8 Advance diffractometer (Bruker AXS, Karlsruhe, Germany) in the range of 5° to 60° with Cu–K radiation at 40 kV.

#### 2.5.4. Surface Morphology Analysis

The fracture surface morphology of SPI-based nanocomposite films was measured by high-resolution and variable pressure scanning electron microscopy (SEM, QUANTA FEG 650, Hillsboro, OR, USA) with 10 kV accelerating voltage.

#### 2.5.5. Opacity Analysis

The optical properties of SPI-based nanocomposite films were characterized by Ultraviolet–visible (UV–Vis) absorption spectra (TU-1901, Purkinje General Instrument Co., Ltd., Beijing, China) in the range of 300–800 nm.

#### 2.5.6. Mechanical Properties

The mechanical properties of SPI-based nanocomposite films, such as tensile strength (TS), Young's modulus (*E*) and elongation at break (EB), were measured with a microcomputer controlled electronic universal testing machine (INSTRON 3365, Norwood, MA, USA) at a testing speed of 50 mm/min as per the report of Li et al. [9]. At least five specimens (80 mm × 10 mm) were measured to calculate the average value.

#### 2.5.7. Contact Angle Measurements

The surface hydrophobicity of nanocomposite films was measured by a contact angle meter (OCA20, DataPhysics Instruments GmbH, Filderstadt, Germany). A drop of deionized water (3 μL) was dropped onto the surface of each film. The contact angle of each drop was recorded at intervals of 0.1s for 120 s.

#### 2.5.8. Water v Vapor Permeability

The water vapor permeability (WVP) of nanocomposite films was determined by a water vapor permeability tester (TSY–T1, Labthink Instruments, Jinan, China) according to the standard method of ASTM E96. All samples were placed into a desiccator at 25 °C and 50% RH with saturated K_2_CO_3_ solution before test. WVP was calculated as follows:

WVP = WVTR*x*/[*P*_0_ (RH_1_− RH_2_)] (1)

where WVP was the water vapor permeability (10^−1^ g·m^−1^·h^−1^·Pa^−1^); WVTR was the water vapor transmission rate (g·m^−2^·24·h^−1^); *P*_0_ was the water vapor pressure (25 °C, 3.169 kPa); (RH_1_−RH_2_) was the relative humidity of the two sides of the film.

#### 2.5.9. Moisture Content (MC), Total Soluble Matter (TSM) and Water Uptake (WU) Tests

All film specimens were placed into a desiccator (25 °C and 50% RH) with saturated K_2_CO_3_ solution before test. Each sample was weighed (*m*_0_) and dried in an air-circulating oven at 103 °C for 24 h. Then, the specimens were weighed again (*m*_1_). MC was calculated as follows:

MC (%) = (*m*_0_ − *m*_1_)/*m*_0_ × 100 (2)

After that, specimens were immersed in beakers with deionized water (50 mL) at 25 °C for 24 h. Then, the samples were dried in an oven at 103 °C for 24 h and weighed (*m*_2_). TSM was calculated as follows:

TSM (%) = (*m*_1_ − *m*_2_)/*m*_1_ × 100 (3)

Before WU test, film samples were dried in desiccators (0% relative humidity) with P_2_O_5_ desiccant at 25 °C for 48 h and the initial weight (*m*_3_) was recorded. Then, specimens were immersed in beakers with deionized water (50 mL) at 25 °C for 24 h and weighed (*m*_4_). WU was calculated as follows:

WU (%) = (*m*_4_ − *m*_3_)/*m*_3_ × 100 (4)

#### 2.5.10. Thermo-Gravimetric Analysis

Thermo-gravimetric analysis (TGA) was obtained with a TGA Q50 (TA instruments, New Castle, DE, USA) under a nitrogen flow of 50 mL/min. The samples were tested from room temperature to 600 °C at a heating rate of 10 °C /min.

## 3. Results and Discussion

### 3.1. Characterization of Graphene, CNC and PEI-Modified CNC

**Figure 1:**
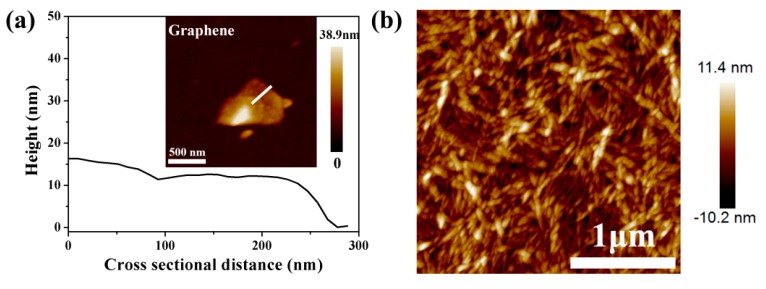

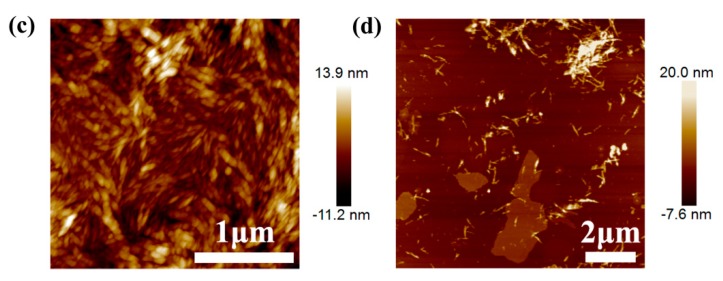
(**a**) AFM height image and cross-section profile of graphene sheets; AFM height images of (**b**) cellulose nanocrystals (CNC), (**c**) PCNC, and (**d**) graphene modified with PCNC.

Atomic force microscopy (AFM) images of the graphene sheets, CNC, and PEI-modified CNC are shown in Figure 1. Graphene dispersion was prepared from the exfoliation and fragmentation of graphite by sonication in the aqueous solutions of BSA. Figure 1a demonstrates the presence of isolated graphene flakes, which had a lateral dimension about 500 nm. The height-profile image revealed that the thickness of each graphene layer was approximately 5 nm and most of the graphene sheets consisted of a few layers. The relatively high parts of graphene sheets were attributed to the adsorbed BSA on their surfaces. As shown in Figure 1b, the pristine CNC was irregularly dispersed in the solution. The rod-like fragments could be found isolated or aggregated with a thickness less than 21.6 nm. The bundles of CNC might be attributed to the strong hydrogen bonds and high specific area between the cellulose nanoparticles. Figure 1c shows the dispersion of PEI-modified CNC nanoparticles with a thickness less than 25.1 nm. These results indicated that the thickness of PEI-modified CNC might be larger than that of pristine CNC, which could be caused by the adsorption of PEI on the CNC surfaces. In addition, the uniform dispersion of PEI modified CNC over the surface of graphene is observed in Figure 1d, which indicates the dramatic improvement of the dispersion and interactions of PEI–CNC with graphene. The fairly regular surfaces of graphene sheets indicated the great compatibility and strong interactions with PEI-modified CNC.

### 3.2. Zeta Potential Measurements

Zeta potential measures were carried out to investigate the surface charge of CNC and graphene sheets. As shown in Table 1, graphene nanosheets possessed a zeta potential of −30.2 mV, suggesting negative charged surfaces. Moreover, the surface of CNC prepared via a sulfuric acid hydrolysis was negatively charged with a zeta potential of −27.6 mV. PEI polyelectrolytes, forming an amino-rich cationic polymer, had been known to effectively interact with nanoparticles via physical adsorption [26]. The CNC modified with PEI showed a zeta potential of 40.4 mV, indicating its strongly positive surface. This significant charge reversal of the zeta potential values of CNC indicated that the dangling chains of PEI attached to the CNC surface formed a positively charged layer, which facilitated their interactions with negatively charged CNC segments and enhanced the attachment of PEI–CNC to negatively charged graphene sheets.

### 3.3. Structural Analysis

**Figure 2:**
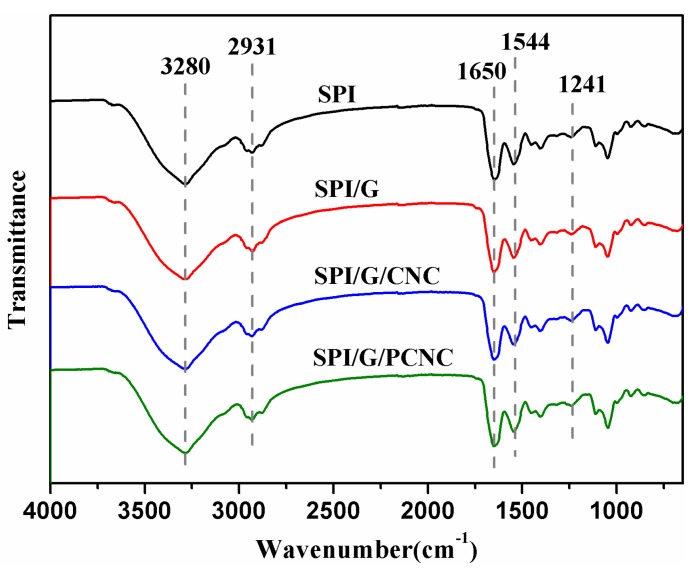
ATR–FTIR spectra of SPI film and SPI-based films modified with graphene, CNC, and PCNC.

ATR–FTIR and XRD were performed to analyze the structural characteristics of the composite films. As shown in Figure 2, the ATR–FTIR spectra of the SPI-based films showed similar characteristic peaks at 1650, 1544, and 1241 cm^−1^, which were attributed to amide I (C=O stretching), amide II (N–H bending), and amide III (C–N and N–H stretching), respectively [27]. The absorption band around 3280 cm^−1^ could be assigned to –OH and N–H stretching vibration, which could form intermolecular hydrogen bonds in the composite [28]. The peak at 2930 cm^−1^ was due to the –CH_2_ groups [29]. The stretching vibrations of C–H and C–O were observed at 1403 and 1046 cm^-1^, respectively [10]. After the modification of graphene and PEI–CNC, the –OH group of the composite film shifted to a higher wave number of 3284 cm^−1^. This feature was probably attributable to the increased hydrogen bonds and polar groups in the polymers, which changed the mode of –OH stretching vibrations [30]. These results indicated that the hydrogen bonding might be an important interfacial interaction in the composite and make a strong connection between filler and polymer molecules to form a continuous film.

**Figure 3:**
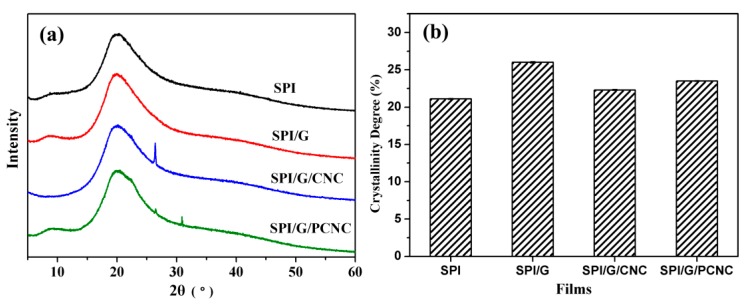
(**a**) XRD diffraction patterns and (**b**) crystallinity of SPI film and SPI-based films modified with graphene, CNC, and PCNC.

The effects of graphene and CNC on the structure conformations of SPI films were analyzed by XRD diffraction patterns (Figure 3a). Both the treated and untreated films exhibited two similar peaks at 2θ = 8.8° and 20.3°, which corresponded to the α-helix and β-sheet structures of the SPI secondary conformation, respectively [31]. The characteristic peak at 2θ = 26.4° was attributed to the (002) diffractions of graphene and graphitic carbon, which confirmed the favorable compatibility and distribution of graphene sheets in the SPI matrix [32]. Compared with the pristine SPI film, these composite films showed a significant increase in the crystallinity degree (Figure 3b), indicating that the structural order of protein conformations was obviously changed. The SPI film modified with graphene exhibited a relatively high crystallinity, suggesting that a higher degree of compact structure of composite film was produced by the addition of graphene [33]. These results indicated that the presence of graphene and CNC might interfere with the integrated structure of SPI and strengthen the molecular association of the film components, allowing for the formation of cross-linking and robust structures [34].

### 3.4. Micromorphology of SPI-Based Nanocomposite Films

**Figure 4:**
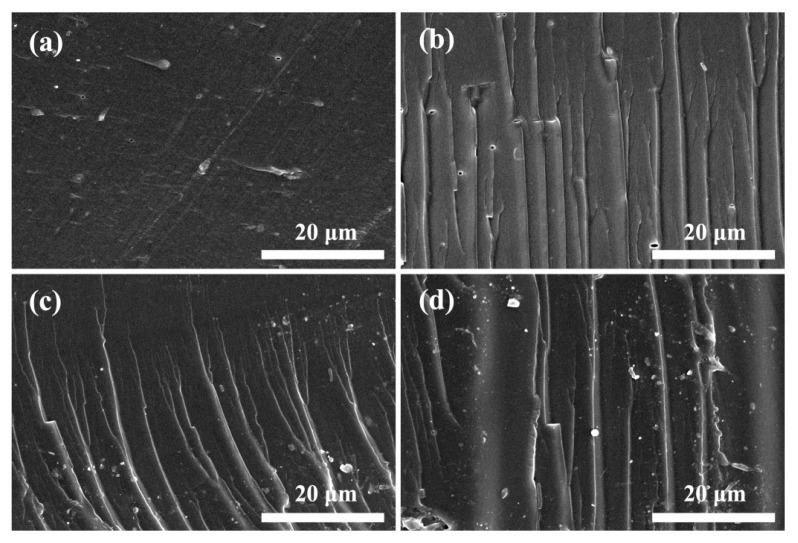
SEM micrographs of SPI film and SPI-based films modified with graphene, CNC, and PCNC.

The fracture surface morphologies of SPI-based nanocomposite films were investigated by SEM observation (Figure 4). As shown in Figure 4a, the pristine SPI film displayed continuous, uniform, and homogeneous structure. Some tiny pores might be due to the evaporation of water during the casting process [35]. In comparison with the control film, the fracture surface of SPI/graphene films exhibited obvious multi-layered structures (Figure 4b), and this was attributed to the addition of graphene. These hierarchical structures were generated intra and inter layers in the films resulting from the corrugated configuration of graphene sheets [23]. After the incorporation of CNC, the cross-section analysis of the nanocomposite films gradually showed some tiny aggregation seemingly embedded in the SPI matrix (Figure 4c), which might be the effective combination of CNC segments [24]. This phenomenon indicated that CNC could be well dispersed within the SPI matrix via excellent interfacial interaction with graphene sheets, which is inconsistent with the report of Pal et al. that completely individualized CNC could act as a dispersing agent for the graphene sheets [25]. As shown in Figure 4d, the cross sectional morphology of SPI/graphene/PCNC film was rougher and the aggregation became even larger, and this is ascribed to the cross-linked network modified with PCNC [36]. This high degree of compact and dense network caused by the presence of long PCNC ridges might contribute to the arrest of sliding and shearing of the laminated structure, resulting in enhanced mechanical properties of SPI-based nanocomposite films [20].

### 3.5. Opacity Analysis

**Figure 5:**
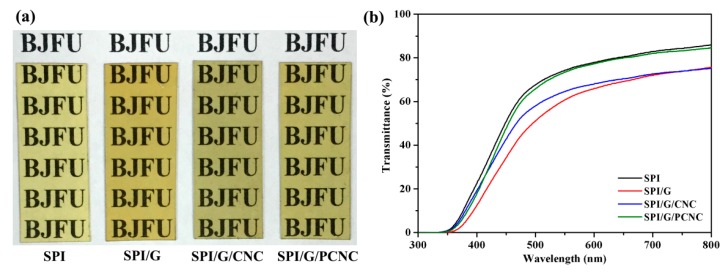
(**a**) Appearance photographs and (**b**) UV–Vis transmittance spectra of SPI film and SPI-based films modified with graphene, CNC, and PCNC.

The optical properties of different composite films were analyzed by appearance photographs and Ultraviolet–visible (UV–Vis) transmittance spectra. As shown in Figure 5a, all SPI-based films appeared to be transparent and yellowish. The untreated SPI film exhibited a relatively light color. With the addition of graphene and CNC, these films became less transparent and more opaque. These minor color changes of the composite films were due to the homogeneous distribution of graphene and CNC in the SPI matrix [37]. The optical transmittance was conducted by UV−Vis spectroscopy to further study the light barrier properties of different composite films. As shown in Figure 5b, the optical transmittance of the SPI-based films gradually decreased with the addition of graphene and CNC. This phenomenon could be explained by the light-scattering and absorption of graphene layers, and this is in good agreement with the report by Lee et al. [38]. The graphene sheets dispersed uniformly and separately in the SPI matrix. This is attributed to the hydrogen bonding interactions between graphene and CNC and interfacial cross-linking in the SPI system [39]. This result verified that the assembly of graphene and CNC enhanced the UV and visible light barrier abilities of SPI-based films, resulting in excellent optical properties, which are necessary for various optoelectronic applications.

### 3.6. Mechanical Properties of SPI-Based Nanocomposite Film

The mechanical behavior of the SPI-based nanocomposite films were evaluated using tensile tests. The results for the tensile strength (TS), Young's modulus (*E*), and elongation at break (EB) are summarized in Table 2. In general, the pristine SPI film exhibited a relatively poor mechanical property with the TS and E values of 3.75 and 77.44 MPa, respectively, which is due to the relatively weak phase interfacial adhesion. With the incorporation of graphene, the TS and E of the SPI/graphene film were clearly increased to 5.89 and 94.30 MPa. This remarkable enhancement in the tensile strength might be attributed to the fact that graphene as the filler in the SPI matrix had significant effects on the mechanical properties of the polymer films [40]. Because of its high specific surface area, graphene can facilitate effective stress redistribution and provide more interfacial areas for load transfer [41]. In addition, the interface strength of the nanocomposite films could be enhanced through π–π interaction between adjacent graphene nanosheets [20]. In particular, with the addition of graphene and PCNC, the TS and *E* of SPI/graphene/PCNC film simultaneously reached even higher values of 7.49 and 97.62 MPa, increases of 99.73 and 26.06%, respectively, compared with that of the control film. This significant reinforcement of the mechanical performance is mainly attributed to the strong synergistic effects of 2D graphene and 1D PCNC in artificial nacre structures through ionic interactions and hydrogen bonds [35]. The strong ionic interactions in the nanocomposites could be achieved by the combination of positively charged PCNC and negatively charged graphene sheets in the laminar structures. Moreover, the hydrogen bonding was also a critical factor to enhance the interfacial interactions and cohesion force in the cross-linked composite films [42], and this is consistent with the XRD results.

Compared with the SPI/graphene film, the EB value of SPI/graphene/CNC film showed a slight increase from 74.92% to 105.83%, suggesting the improved flexibility of the composite film. This phenomenon was probably caused by the fact that CNC could serve as the dispersing agent during graphene exfoliation in water and graphene sheets were well CNC-assisted dispersed in the SPI matrix [43]. The homogeneous dispersion and strong interfacial interactions between graphene and CNC could lead to a significant improvement on mechanical behaviors of the SPI-based films. These results demonstrated that the mechanical performance of the SPI-based composite films can be significantly reinforced by the combination of graphene and PCNC.

### 3.7. Surface Hydrophilicity and Water Resistance of SPI-Based Nanocomposite Films

**Figure 6:**
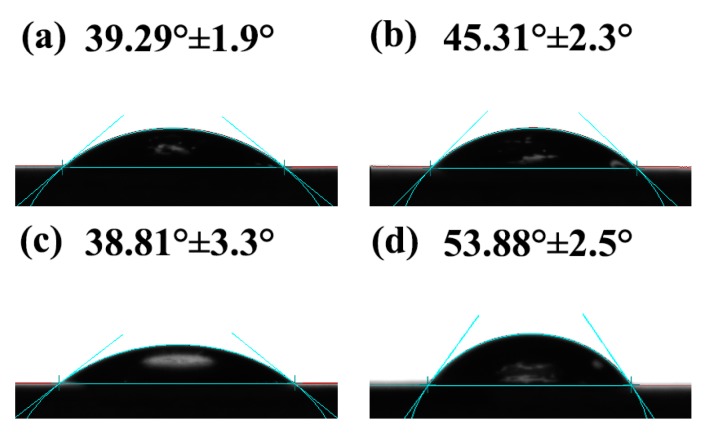
Water contact angles (WCA) of SPI film and SPI-based films modified with graphene, CNC, and PCNC.

The surface hydrophilicity of SPI-based nanocomposite films was evaluated by measuring the water contact angles (WCA). As displayed in Figure 6, the untreated SPI film exhibited a relatively low WCA, which indicated its high hydrophilic property. This feature might be caused because SPI contains many polar amino acids, which resulted in a film that was more susceptible to high moisture [44]. In comparison with the control film, the WCA value of the SPI/graphene film was clearly increased from 39.29° to 45.31°, suggesting that the hydrophobic graphene layer was successfully transferred into the SPI substrate [45]. With the addition of graphene and PCNC, the WCA value of SPI-based nanocomposite film significantly increased to 53.88°, which might be attributed to the effects of hydrogen bonds and polar groups in the composite [40]. These results confirmed the favorable surface hydrophobicity of the SPI-based nanocomposite film modified with graphene and PCNC.

The water vapor barrier abilities of the nanocomposite films were analyzed by water vapor permeability (WVP). As summarized in Table 3, the WVP of the SPI/graphene film was significantly reduced as a result of the incorporation of functionalized graphene sheets, suggesting the superior effect of graphene on the permeability of the composite, which was consistent with previous studies of Hocker et al. [42]. In terms of barrier properties of food package materials, SPI/graphene /PCNC film exhibited a higher WVP value compared to that of whey protein isolate films [1].This significant reduction in the permeability of the SPI-based films modified with graphene might be attributed to the tortuosity effect induced by the high aspect ratio of graphene and the reduced mobility of the polymer chains [35]. The reduction in the chain mobility could result from the effect of the graphene and PCNC on the crystalline and phase transitions, which could be described as the interactions of the nanoparticles with the polymer chains, resulting in a region of constrained SPI chains mobility [3]. Therefore the SPI/graphene/PCNC film exhibited enhanced water vapor barrier performance, which is favorable for food packaging applications.

The effects of graphene and CNC on the water resistance of the SPI-based nanocomposite films could be investigated by moisture content (MC), total soluble matter (TSM), and water uptake (WU). As listed in Table 3, the untreated SPI film exhibited poor water resistance with relatively high values of MC, TSM and WU. With the incorporation of graphene and PCNC, the SPI-based nanocomposite films did not show obvious changes in the value of TSM. However, compared with the control film, the MC and WU of SPI/graphene/PCNC film were significantly decreased by 24.54% and 47.18%, respectively. These results could be attributed to the presence of enhanced hydrogen bonds between the filler and SPI matrix, which decreased the diffusion speed of water molecules into the cross-linked structure [43]. In addition, the tortuosity effect of the relatively dense and integrated structure could prevent the SPI matrix from swelling under high moisture conditions, which resulted in the superior water resistance of the nanocomposite film [34].

### 3.8. Thermal Stabilities of SPI-Based Nanocomposite Films

**Figure 7:**
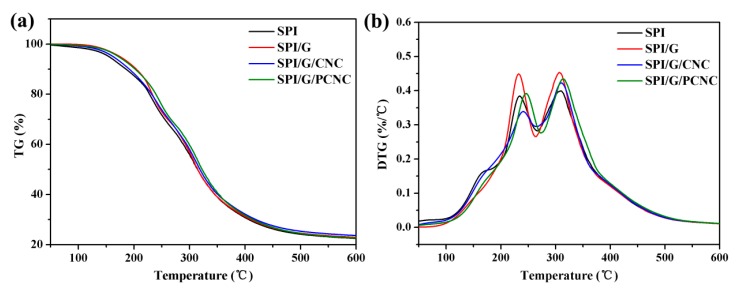
(**a**) Thermo gravimetric (TG) and (**b**) derivative thermo gravimetric (DTG) curves of SPI film and SPI-based films modified with graphene, CNC, and PCNC.

The thermal properties of the SPI-based nanocomposite films were investigated by Thermo gravimetric (TG) and derivative thermo gravimetric (DTG) analysis (Figure 7). The thermal degradation data of the SPI-based nanocomposite films are summarized in Table 4. As shown in Figure 7a, the SPI-based nanocomposite films generally possessed three main degradation stages. The initial weight loss from 50 to 130 °C was associated with the evaporation of absorbed moisture in the SPI-based films. The second stage from 130 to 270 °C was attributable to the evaporation and degradation of glycerol. In the third stage from 270 to 450 °C, the weight loss was primarily due to the thermal degradation of the SPI matrix [44]. The TG curves of SPI/graphene/PCNC film shifted slightly to a higher temperature than the control film, which indicates the improvement of thermal stability. As listed in Table 4, compared with the control film, the initial degradation temperature (*T_i1_*) of the film modified with PCNC clearly increased from 143.48 to 155.06 °C, which might be attributed to the presence of abundant hydrogen bonds in the composite that prevent the evaporation of absorbed moisture in the films [2]. In addition, the temperature at maximum degradation rate (*T*_max_) significantly increased with the incorporation of graphene and PCNC, which was associated with the cross-linking and multiple interfacial interactions between filler and polymer molecules, resulting in the enhanced thermal stabilities of the SPI-based nanocomposite films [4]. As a carbon-based filler, graphene was considered the most promising thermally conductive filler in polymer composites because of the combination of high thermal conductivity and large aspect ratios [18]. Therefore the sheet-like geometry of graphene might have higher interfacial thermal resistance and promote the improvements in the thermal stabilities of the SPI-based nanocomposite films.

## 4. Conclusions

In conclusion, we have proposed a facile and green approach for the preparation of stable graphene dispersion and fabricated the SPI-based nanocomposite film via the combination of 2D negative charged graphene and 1D positive charged PEI-modified CNC. The surface morphologies of graphene sheets and CNC segments were investigated by AFM. The Zeta potential measurements confirmed the surface charge of different components. In addition, the structural characterization of the composite films was analyzed by ATR–FTIR and XRD patterns, which showed the increased hydrogen bonds and multiple interface interactions between the filler and SPI matrix. The cross-linked and laminated structures were observed by SEM images. In comparison with the control film, the TS of SPI/graphene/PCNC film increased from 3.75 to 7.49 MPa, and the WCA increased from 39.29° to 53.88°, which indicated the significantly enhanced mechanical property and surface hydrophobicity of this nanocomposite film. Moreover, the UV–visible light barrier ability, water resistance, and thermal stability were also obviously improved. Therefore, these high performance SPI-based nanocomposite films modified with graphene and PCNC could be valuable for potential applications in food packaging materials.

## Figures and Tables

**Table 1:** Zeta potential of CNC, PCNC, and graphene.

Samples	Zeta Potential (mV)
CNC	−27.6 (0.21)
PCNC	40.4 (0.45)
graphene	−30.2 (0.24)

The values in parenthesis were the standard deviations.

**Table 2:** Thickness, tensile strength (TS), Young's modulus (E), and elongation at break (EB) of SPI film and SPI-based films modified with graphene, CNC, and PCNC.

Films	Thickness (mm)	TS (MPa)	*E* (MPa)	EB (%)
SPI	0.256 (0.014)	3.75 (0.34)	77.44 (3.39)	173.69 (0.14)
SPI/graphene	0.293 (0.010)	5.89 (0.47)	94.30 (4.23)	74.92 (0.17)
SPI/graphene/CNC	0.289 (0.011)	6.26 (0.29)	82.04 (3.85)	105.83 (0.13)
SPI/graphene/PCNC	0.263 (0.010)	7.49 (0.30)	97.62 (5.43)	87.14 (0.06)

The values in parenthesis are the standard deviations.

**Table 3:** Water vapor permeability (WVP), moisture content (MC), total soluble matter (TSM), and water uptake (WU) of SPI film and SPI-based films modified with graphene, CNC, and PCNC.

Samples	WVP (10^−1^·g m^−1^·h^−1^·Pa^−1^)	MC (%)	TSM (%)	WU (%)
SPI	11.04 (0.37)	17.89 (0.8)	34.16 (1.9)	244.90 (7.4)
SPI/graphene	9.29 (0.24)	11.84 (1.9)	33.59 (0.9)	167.01 (4.8)
SPI/graphene/CNC	10.30 (0.13)	14.07 (1.7)	33.79 (1.0)	184.11 (6.1)
SPI/graphene/PCNC	9.55 (0.35)	13.50 (0.5)	33.06 (1.8)	129.35 (5.4)

The values in parenthesis were the standard deviations.

**Table 4:** Thermal degradation data of SPI film and SPI-based films modified with graphene, CNC, and PCNC.

Samples	*T*_i1_ (°C)	*T*_max1_ (°C)	*T*_i2_ (°C)	*T*_max2_ (°C)
SPI	143.48	232.91	291.93	307.29
SPI/graphene	148.41	234.19	294.80	308. 89
SPI/graphene/CNC	154.16	240.88	294.79	310.67
SPI/graphene/PCNC	155.06	246.05	295.57	312.83

*T*_i_: initial temperature of degradation; *T*_max_: temperature at maximum degradation rate.
